# From field to plate: 50 years of plant-based food production and emerging risks to planetary and women’s health

**DOI:** 10.3389/fnut.2025.1619844

**Published:** 2025-06-30

**Authors:** Maria Julia Miele, Priscila Pereira Coltri, Camila Ferreira Soares, Renato Teixeira Souza, Rodolfo Carvalho Pacagnella, José Guilherme Cecatti, Barbara Teruel

**Affiliations:** ^1^School of Agricultural Engineering, University of Campinas (UNICAMP), Campinas, Brazil; ^2^Department of Obstetrics and Gynecology, School of Medical Sciences, University of Campinas (UNICAMP), Campinas, Brazil; ^3^Center for Meteorological and Climatic Research Applied to Agriculture (CEPAGRI), University of Campinas, Campinas, Brazil; ^4^Department of Demography, Institute of Philosophy and Human Sciences, University of Campinas (UNICAMP), Campinas, Brazil

**Keywords:** agri-food systems, dietary patterns, planetary health, pesticide residues, reproductive age

## Abstract

**Introduction:**

Planetary health is a comprehensive approach that reveals the complex interconnections between sustainable human and environmental balance. This concept is rooted in the understanding that the health and progress of human civilization depend on thriving natural systems and their preservation. However, the profound impact of plant-based foods on both human and planetary health remains underestimated. Diet has the power to shape present health and compromise the future of entire generations.

**Methods:**

This study analyzed 48 years (1974–2022) of trends in Brazilian agricultural production, focusing on plant-based foods and their potential risks to the environment and the health of women of reproductive age—a particularly vulnerable population. Using national databases, we explored associations between food production and consumption patterns within this demographic.

**Results:**

Although rice and beans are traditionally central to Brazilian diets, production data suggest that the dominant crops available to women of reproductive age are soy and sugarcane. These crops are associated with numerous pesticide authorizations, suggesting high levels of contamination by toxic chemical residues.

**Discussion:**

Our findings suggest that plant-based diets represent a disruptive concept that requires careful revision, given the high contamination by toxic chemical residues in food, water, and humans. Beyond nutrient-based public policies, food management—from field to plate—must integrate food security to ensure a healthy and sustainable food system.

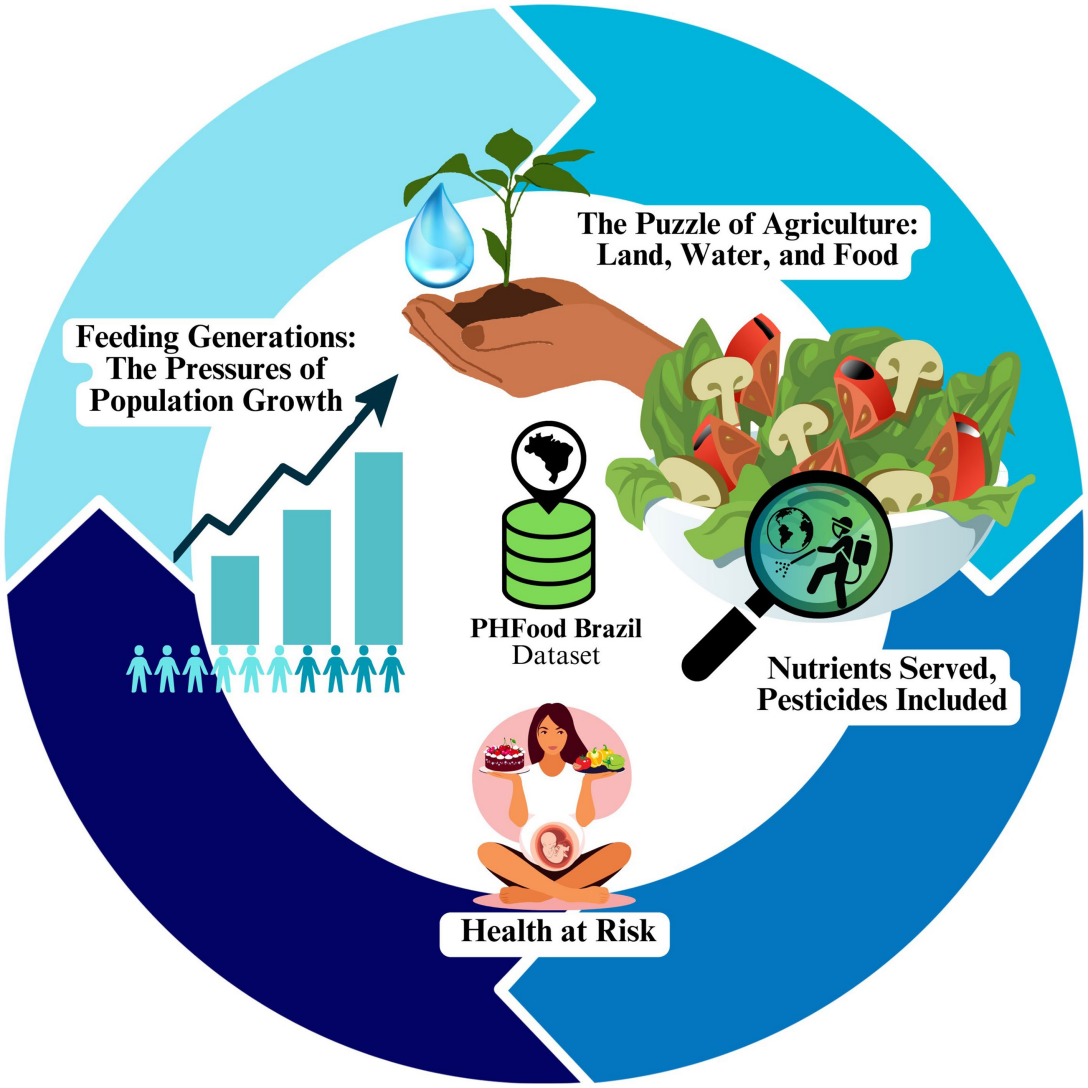

## 1 Introduction

The Planetary Health concept emphasizes the interdependence between human health and the integrity of Earth’s ecosystems (1). It highlights the need for sustainable agricultural practices that ensure productivity while operating within planetary boundaries (2). However, to meet the growing global food demand - projected to exceed 9 billion people by 2050 (3), conventional agricultural production systems have often exceeded sustainable land and water use thresholds.

As one of the world’s leading agricultural powers, Brazil plays a key role in global food production. The expansion of conventional Brazilian agriculture, characterized by high yields per cultivated area, has been driven primarily by the intensified use of agricultural inputs, particularly pesticides and fertilizers (4). In 2018, Brazil sold 550,000 tons of active pesticide ingredients, a figure that rose to 800,000 tons in 2022 (5), surpassing the combined pesticide consumption of China and the United States, making it the world’s largest pesticide consumer (6). This makes Brazil a strategic context for studying the intersection of food production, pesticide exposure, and public health risks, particularly among women of reproductive age, who are biologically more susceptible to these exposures.

The impact of pesticides extends beyond food production, affecting water quality, ecosystem health, and human exposure (7). This exposure directly impacts food consumption, particularly affecting vulnerable groups, such as women of reproductive age. Studies have linked pesticide exposure to an 18% lower likelihood of conception and a 26% higher risk of pregnancy loss (8). Additionally, maternal exposure to pesticides has been associated with an increased risk of preterm birth, low birth weight, and congenital malformations, leading to long-term consequences for child development (9).

Some plant-based diets may pose hidden risks when based on pesticide-intensive monocultures. In this context, it is essential to bridge the gap between food production and consumption, ensuring the protection of future generations through women’s health. This study aims to provide insights for public policies that promote food and nutritional security while prioritizing human health and environmental sustainability.

## 2 Materials and methods

This is a longitudinal, ecological, and exploratory study that tracks food and nutrient production in Brazil, examining its impacts on sustainability through the Big Data *Planetary Health: A Comprehensive View of Food in Brazil* (10). The PHFood Brazil database integrates information spanning nearly five decades (1976–2023), sourced from Brazilian government agencies such as the Ministry of Agriculture and Livestock (MAPA), Municipal Agricultural Production (PAM-IBGE), National Health Surveillance Agency (ANVISA), and the National Water and Basic Sanitation Agency (ANA). The variables used in this study included water use (m^3^), planted area (hectares), agricultural production (tons), nutrients provided by foods, and pesticides registered according to toxicological classification and environmental risk, disaggregated by year, region, state, and age group of women (12 to 49 years).

The data related to agricultural production (PAM) were organized by the Brazilian Institute of Geography and Statistics (IBGE) and provided information on average yield and the production of both temporary and permanent crops at the state and regional levels. Data collection was conducted by IBGE agents, with information sourced from agricultural technicians, producers, and sector-specific entities. For this study, only edible agricultural products were considered, to later align with food groups consumed by the population (Table 1).

**Table 1:** Plantation food groups and corresponding food list used in this study.

Plantation food groups	Food list
Beverages	Coffee, Sugarcane
Cereals	Rice, Oat, Corn, Wheat
Fruits	Avocado, Pineapple, Acai, Banana, Cashew, Persimmon, Coconut, Fig, Guava, Orange, Lime, Papaya, Mango, Passion Fruit, Apple, Watermelon, Melon, Pear, Peach, Tangerine, Grape, Acerola, Plum, Strawberry
Pulses, Seeds, and Nuts	Peanut, Peas, Bean, Soybean
Tubers and Roots	Potato, Cassava, Beetroot, Yam, Carrot, Taro
Vegetables	Garlic, Olive, Onion, Heart of Palm, Tomato, Swiss Chard, Watercress, Lettuce, Kale, Broccoli, Cauliflower, Cabbage, Pumpkin, Zucchini, Eggplant, Chayote, Scarlet Eggplant, Cucumber, Bell Pepper, Okra, Jiló

The variables included harvested areas and production, considering agricultural calendars or extended harvest periods following the methodology established by the PAM technical team (11).

**Figure 1:**
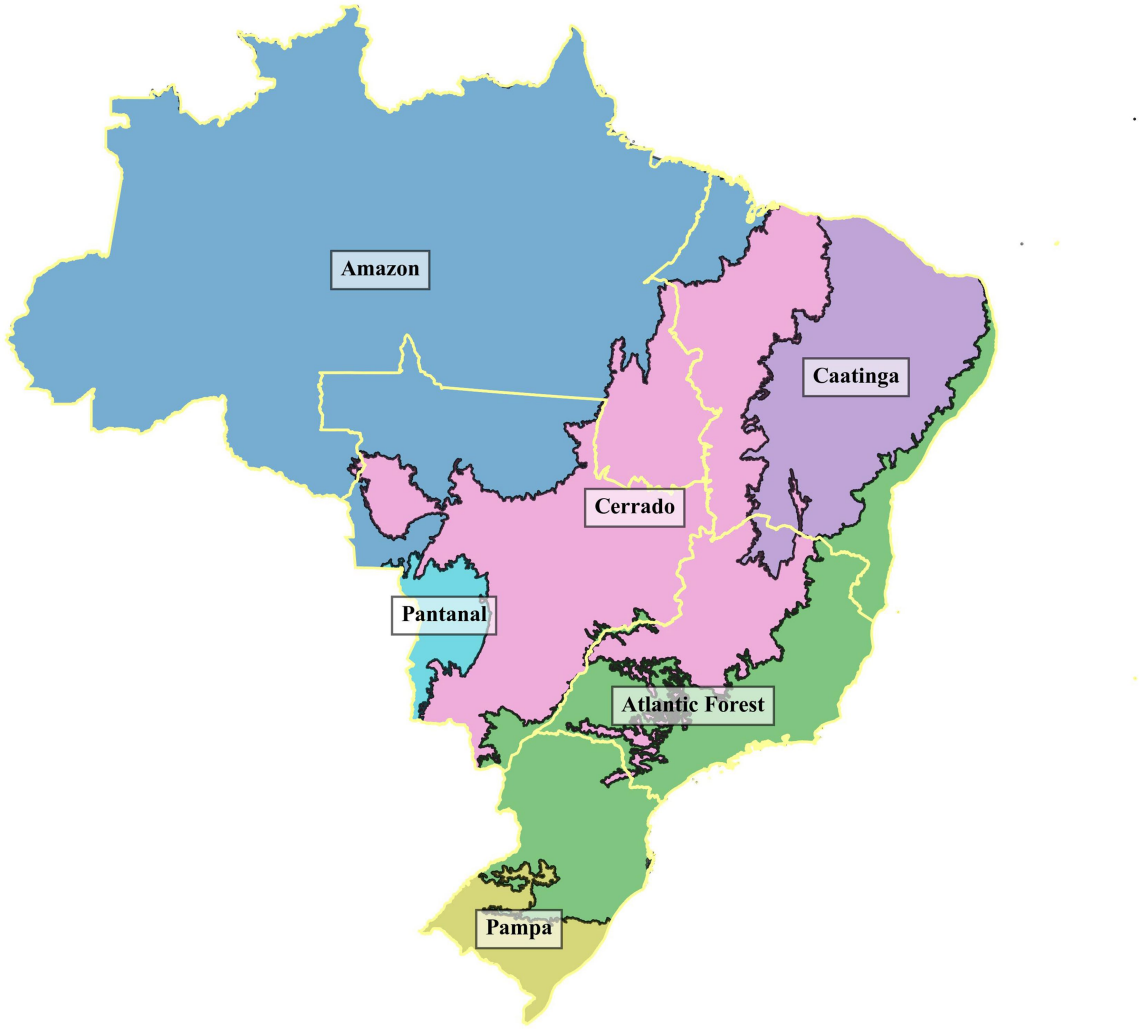
The spatial distribution of Brazil’s biomes. The regional divisions are outlined in light yellow for better visualization. (1) Amazon: Characterized by a dense tropical rainforest and a humid equatorial climate. It has high annual rainfall (2,000–3,500 mm), a warm and stable temperature (25–28°C), and nutrient-poor soils due to intense leaching. (2) Atlantic Forest: The most biodiverse biome, covering the east coast of Brazil. It has a humid climate with high rainfall (1,200–4,000 mm per year), stable and mild temperatures (20–26°C), and consistently high humidity (above 80%). The soil is generally fertile but vulnerable to erosion due to deforestation. (3) Caatinga: A semi-arid biome in northeastern Brazil, composed of drought-tolerant vegetation. It is subject to high climate variability and desertification risks, with low and irregular rainfall (250–800 mm per year), shallow and rocky soils, and high evaporation rates. (4) Cerrado: A tropical savanna in central Brazil. It has a pronounced dry and rainy season, with annual precipitation between 800 and 2,000 mm. The soil is generally acidic and nutrient-poor, requiring liming and fertilization for intensive agriculture. (5) Pampa: A temperate grassland located in southern Brazil. It has annual rainfall between 1,200–1,500 mm, fertile soils, and is primarily used for livestock grazing and pasturelands. (6) Pantanal: The largest tropical wetland in the world. Rainfall varies from 1,000 to 1,500 mm per year, with poorly drained soils that retain water, allowing the coexistence of aquatic and terrestrial ecosystems. It is an important area for livestock farming and wildlife conservation. Sources: Figure created using shapefiles from the Brazilian Institute of Geography and Statistics (IBGE): Biomas_250mil and BRUF2023. Source: https://www.ibge.gov.br/geociencias/downloads-geociencias.html. licensed under the Public Domain.

Brazil has the world’s greatest diversity of climates and biomes, which play a crucial role in agricultural production. Figure 1 presents the biodiversity map of Brazilian biomes across different regions.

The information related to water use in agricultural fields was obtained from the National Water and Basic Sanitation Agency (ANA) (12). The estimation of water demand for rainfed agriculture was based on the calculation of the water balance of cultivated areas, attributing to rainfed consumption the fraction of water required by crops that are supplied by natural sources, such as precipitation and soil moisture. The data included water demands related to crop water requirements, actual water consumption, and water deficits for both temporary and permanent crops (2013 to 2017).

Pesticide registration data were obtained from the Ministry of Agriculture, Livestock, and Supply (MAPA) – AGROFIT database (13) based on product codes linked to their active chemical ingredients. Exclusion criteria during data processing eliminated duplicates where the year, product code, chemical ingredient, environmental classifications, type of use, toxicity levels, and associated foods were identical.

Toxicity and environmental risk classifications followed the guidelines established by the National Health Surveillance Agency (ANVISA) (14, 15). This revision expanded the toxicity categories classified as follows: Class 1 Extremely Toxic: high potential to cause acute harm to human health; Class 2 Highly Toxic: elevated risk of acute toxicity; Class 3 Moderately Toxic: moderate potential for acute toxicity; Class 4 Slightly Toxic: low potential to cause acute harm; Class 5 Unlikely to Cause Acute Harm: products considered unlikely to provoke significant acute toxic effects. This classification considers criteria such as toxicity to non-target organisms (including bees, fish, and soil microorganisms), environmental persistence, bioaccumulation potential, and mobility in soil and water.

Pesticides are also categorized according to different levels of environmental impact: (1) Very Hazardous: high potential to cause significant harm to ecosystems; (2) Hazardous: moderate to high potential to negatively affect the environment; (3) Slightly Hazardous: limited environmental impact; (4) Unlikely to Cause Harm: low toxicity to non-target organisms and minimal environmental impact. Both the reclassification of human toxicity and environmental risk are aligned with the Globally Harmonized System of Classification and Labeling of Chemicals (GHS) (16). The data related to agricultural variables are presented in Table 2.

**Table 2:** Description of agricultural variables used in the study, covering production, water use, and pesticide applications.

Variable	Unique values	Available data	Description
Year	49	438,320	Year of data collection
Region	5	438,273	Geographic region
Food	60	436,124	Food name
Harvest Area (ha)	14,176	432,688	Harvested area in hectares
Production (tons)	22,683	432,688	Food production in tons
Water Need	709	24,965	Water requirement for production
Water Use	709	24,965	Water usage for crops
Water Deficit	706	24,965	Water deficit indicator
Code	1700	406,199	Pesticide Identification Number
Pesticide Commercial Name	1700	406,199	Commercial name of the pesticide
Pesticide	445	406,199	Active ingredient of the pesticide
Pesticide Class	8	120,524	Pesticide classification
Organic Status	2	406,199	Organic certification status
Toxicity Class	5	406,199	Toxicity classification of the pesticide
Environmental Impact	4	406,199	Environmental impact level
Authorized Use	1	120,524	Authorization status for use
Max Residue Limit (MRL)	31	120,524	Maximum Residue Limit allowed
Acceptable Daily Intake (ADI)	24	120,524	Acceptable Daily Intake for humans
Residue Percentage	59	120,524	Percentage of pesticide residue in food
Food Acquisition (kg)	2,284	157,218	Household food acquisition in kg
Plantation Group	7	438,320	Classification of crops (e.g., fruits, grains)

To understand dietary habits, data from the Household Budget Survey (Pesquisa de Orçamentos Familiares, POF) conducted by the Brazilian Institute of Geography and Statistics (IBGE) were analyzed for the years 2008 and 2018. This study utilized the 5th and 6th editions of the POF, both conducted by IBGE, focusing on the average dietary intake of women of reproductive age. Both surveys applied a two-stage cluster sampling design to ensure socioeconomic and geographic representativeness. Food consumption data were collected from sub-samples of households, including all individuals aged 10 years or older. The final samples comprised 34,003 individuals in 2008 and 46,164 in 2018. The sampling in both studies included both rural and urban areas, based on the administrative criteria established by Brazilian municipalities. Exclusion criteria included both animal-based foods and products not derived from agricultural production. To assess the volume of food consumed, we considered the traditional Brazilian preparation known as “*media,”* composed of equal parts milk and coffee, labeled as *CAFÉ COM LEITE* or *CAFÉ COM LEITE, NÃO DETERMINADO*.

To calculate de nutrient intake, was used the *Brazilian Food Composition Table* (TBCA), developed by the University of São Paulo (USP) and the *Food Research Center* (FoRC), version 7.2, published in São Paulo in 2022 and accessed on 10/02/2024. The two harmonized datasets from the *Household Budget Survey* (Pesquisa de Orçamentos Familiares - POF) were compiled from the FAO/WHO GIFT (Global Individual Food Consumption Data Tool) (17) From these datasets, we selected the final sample based on the food consumption patterns of the study’s target population - women of reproductive age (15 to 49 years). This definition was chosen to assess the potential risks associated with the consumption of pesticide residues, that may be present in plant-based diets among this population.

The classification of food groups in this study was guided by the structure of the nineteen food groups from the FAO/WHO GIFT platform, which is based on the FoodEx2 system developed by the European Food Safety Authority (EFSA) and adapted for global use in collaboration with FAO and WHO (18). This study did not assess food groups of animal origin, such as *Milk and milk products*, *Eggs*, *Fish*, *Meat*, and *Insects and grubs*. To align with the primary objective, which is the evaluation of agricultural products in comparison to the quality of dietary consumption in Brazil, we also excluded food groups that include *composite dishes*, processed foods, or products intended for specific supplementation.

Methodological procedures and potential data limitations are summarized in the codebook published with the PHFood Brazil dataset, available in the repository to support transparency and reproducibility (10).

### 2.1 Statistical analysis

Numerical variables are presented as means and standard deviations, while categorical variables are represented by absolute numbers and percentages. In the descriptive statistics, values presented in the graphs were rescaled to improve readability. To compare differences in food group consumption across regions and the classifications of pesticide risk and environmental impact, Kruskal-Wallis tests were applied for numerical variables, such as average food group consumption and average crop yield (in tons) or planting area (in hectares). Chi-square tests were used for categorical variables, such as pesticide toxicity classifications and environmental impact ratings. Results were considered statistically significant when the *p*-value was less than 0.05.

Data analysis was performed using R (version 4.3.3) for data mining, variable organization, clustering, and graphical presentation (19). Python (version 3.12.8) was used for spatial data processing and biome distribution mapping, employing the GeoPandas and Matplotlib libraries for visualization (20).

This study exclusively utilized publicly available datasets from open-access platforms, ensuring full compliance with ethical and legal standards. No personally identifiable information or sensitive data were accessed, analyzed, or disclosed. All datasets adhere to the FAIR principles (Findability, Accessibility, Interoperability, and Reusability), ensuring transparency and reproducibility. The research followed the ethical guidelines outlined in the Helsinki Declaration (2013), which establishes principles for conducting research with integrity, respect for human rights, and data protection. Given the nature of the study, no institutional ethics approval was required.

## 3 Results

The food items were classified based on their availability in the databases presented here and considering their nutritional characteristics and dietary consumption patterns (Table 1).

### 3.1 Agricultural production

**Figure 2:**
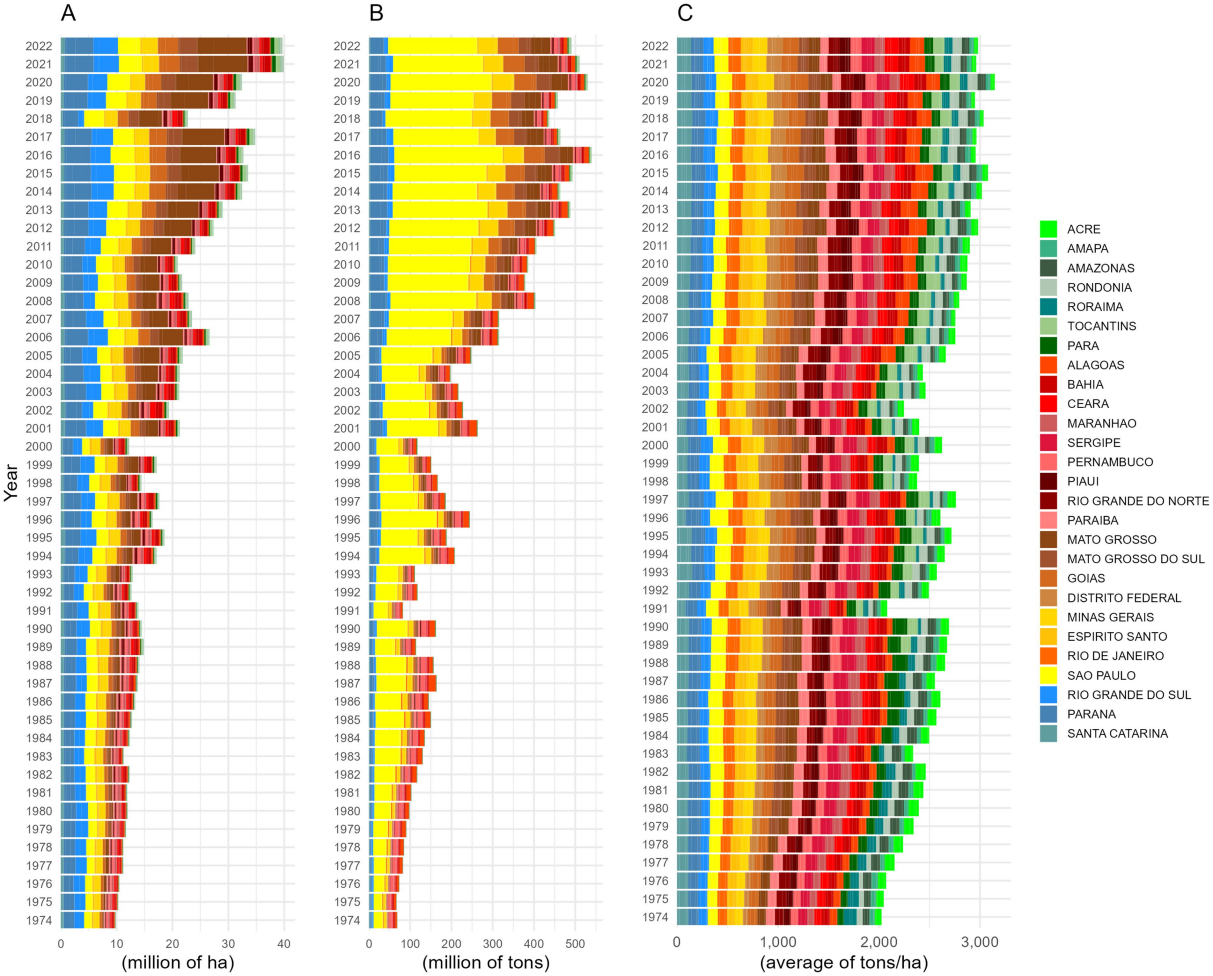
Analysis of agricultural trends by state over the years. This figure presents an analysis of agricultural trends across states, with a region-based color palette distinguishing regions of the country: North (Green), Northeast (Red), Central-West (Brown), Southeast (Yellow), and South (Blue). The panels are organized as follows: **(A)** shows the average harvest area (millions of hectares), illustrating trends in the scale of land use; **(B)** represents the average total food production (millions of tons), revealing variations in agricultural output over time; **(C)** displays the average food production per hectare, highlighting the efficiency of agricultural land use.

Figure 2 presents an analysis of agricultural production by federal unit and region of Brazil.

Figure 2A presents the average harvested area (in hectares), highlighting the crop groups that occupied the largest cultivated areas in each region. The analysis reveals that, with exceptionally the Southeast, the “Pulses, seeds, and nuts” group had the most extensive harvested areas in all other regions between 2021 and 2022. In 2021, the Central-West recorded the largest harvested area, totaling 2.6 million hectares, followed by the South with 2.4 million hectares and the Northeast with 271 thousand hectares. In 2022, the North registered 205 thousand hectares for the same crop group. The Southeast, showed the largest harvested area in 2016, primarily allocated to crops used for beverage production, reaching 912 thousand hectares.

Figure 2B presents the highest average food production (in tons) recorded across Brazil’s regions. The 2016 was a key year for agricultural output, particularly in the beverages group registered the highest production volumes in the Southeast (60.6 million tons), Central-West (20.7 million tons), and South (9.46 million tons). In the Northeast, the highest production of the “beverages” group was recorded in 1987, reaching 4.45 million tons. In contrast, the North region exhibited a different production pattern, with the highest average yield occurring in 2022, in the Pulses, seeds, and nuts group, totaling 659 thousand tons.

Figure 2C presents the variation in agricultural productivity (tons per hectare) across Brazil’s regions. The highest productivity values were observed in distinct years and crop groups. In 2018, the Southeast recorded the highest yield, reaching 63.51 tons per hectare for vegetables and their products. In 1995, the South registered 60.02 tons per hectare for fruit production, while in 2020, the Central-West had the highest yield, with 56.69 tons per hectare for vegetables and their products. The Northeast and North showed the highest fruit production in 1988, with 47.49 and 46.29 tons per hectare, respectively.

In the analysis of food production relative to cultivated area, soybeans stood out as the most significant crop over the 48 years evaluated. Between 1974 and 2000, the Southern region dominated soybean cultivation, with planted areas ranging from a minimum of 2.5 million hectares to a maximum of 4.0 million hectares. From 2001 onward, the state of Mato Grosso became the leading soybean producer, with cultivated areas varying between 3.1 million hectares and 11 million hectares.

Whereas considering total annual food production in tons, São Paulo emerged as the largest individual producer throughout the 1974–2022 period, with sugarcane as its main crop. The food production ranged from 35.6 million tons (1975) at its lowest to 450 million tons at its peak (2017). However, evaluating production (in tons) by region and year, sugarcane was the crop with the highest volumes for all regions, except in the North. The recorded yields of sugarcane were in 2017 in the Southeast (450 million tons in São Paulo), followed by the Center-West in 2020 (75.9 million tons in Goiás), the South in 2009 (53.8 million tons in Paraná), and the Northeast in 1987 (34.5 million tons in Alagoas). The North exhibited a distinct pattern, with its highest production recorded in 2007 when cassava reached 5.2 million tons in the state of Pará.

In Brazil, certain agricultural commodities are closely aligned with traditional dietary habits, particularly staple crops such as rice and beans. Throughout most of the historical period analyzed, rice production generally exceeded that of beans; however, in 1997, the Northeast was an exception, where bean cultivation surpassed rice production, with an average of 807 thousand hectares dedicated to beans.

Regarding regional commodity production per cultivated area, the Central-West recorded the highest average in 1977, with 1.5 million hectares planted, followed by the South in 2011 (1.2 million hectares), the Northeast in 1982 (1.1 million hectares), the Southeast in 1976 (852 thousand hectares), and the North in 1989 (381 thousand hectares). For bean cultivation, the largest planted area per hectare was observed in the Northeast in 1988, reaching 898 thousand hectares, followed by the North in 1982 (880 thousand hectares), Southeast in 1974 (849 thousand hectares), Central-West in 2018 (263 thousand hectares) and North in 1994 (166 thousand hectares).

### 3.2 Water resources and agricultural demand

The highest water consumption per crop was recorded for sugarcane in the state of São Paulo, with an average annual flow measured in millions of cubic meters per second. The five years with the highest water use, in decreasing order, were 2016, 2015, 2014, 2017, and 2013. Then, soybeans in Mato Grosso were classified as the second crop with the highest water consumption, with peak use recorded in four consecutive years (2017, 2015, 2016, and 2014).

**Figure 3:**
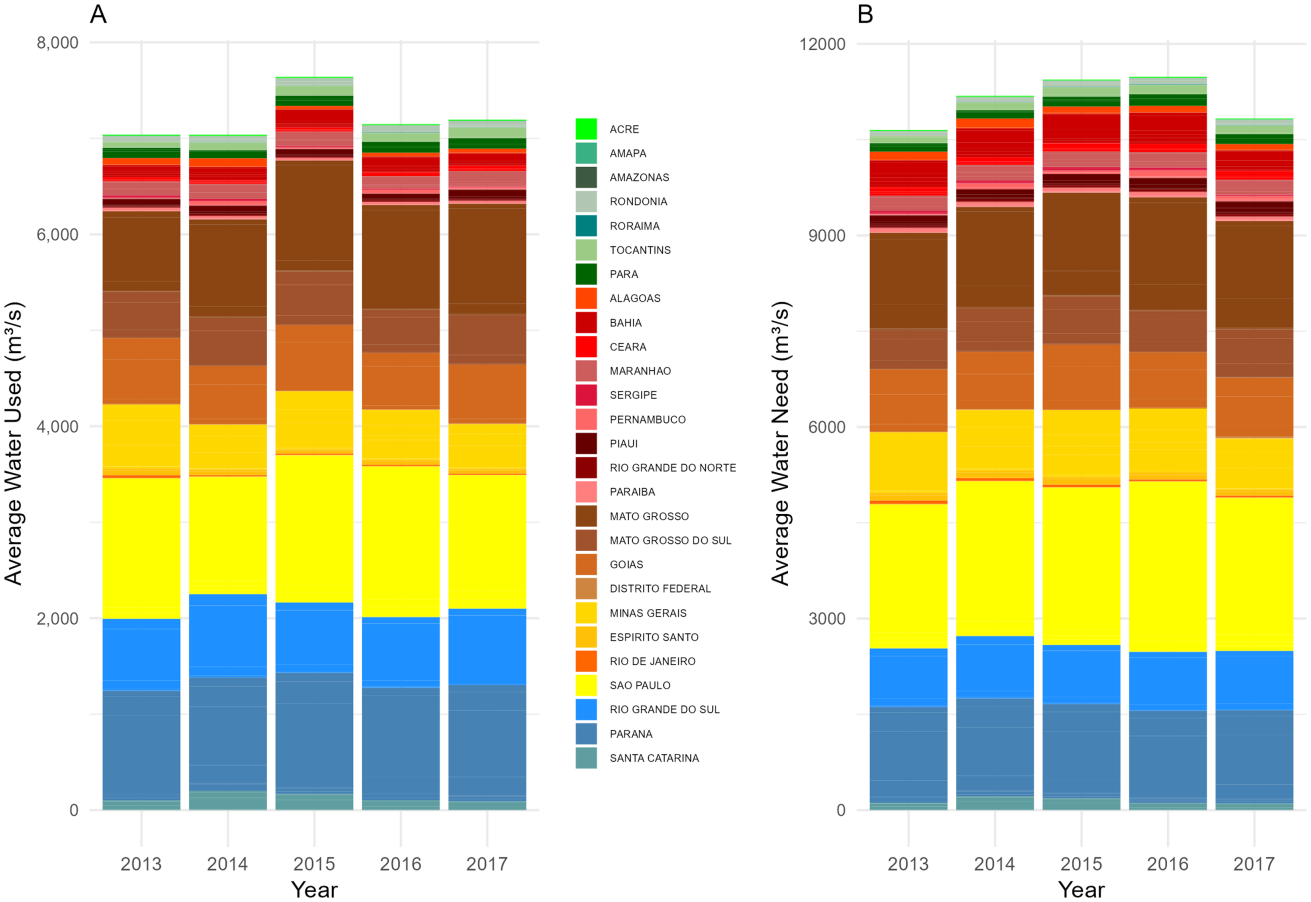
Annual average water requirements by Brazilian states. The color palette differentiates states according to their regions: North - Green, Northeast - Red, Central-West - Brown, Southeast - Yellow, and South - Blue. **(A)** Water used: Represents the effective precipitation consumed, defined as the portion of precipitation that directly reaches the soil, infiltrates, and becomes available to plants (cubic meters per second, annual average). **(B)** Water Need: Refers to the actual crop evapotranspiration, indicating the amount of water required for the optimal development of the crop(s) (cubic meters per second, annual average).

The water deficit percentage, which reflects the gap between water demand and actual water use, ranged from 204.43 to 232.59% in São Paulo and from 31.53 to 20.88% in Mato Grosso, based on the availability of green water for crops (annual average percentage). The highest water deficit percentage was recorded for corn in Minas Gerais in 2016, reaching 498.44% (Figure 3).

### 3.3 Nutrients produced

**Figure 4:**
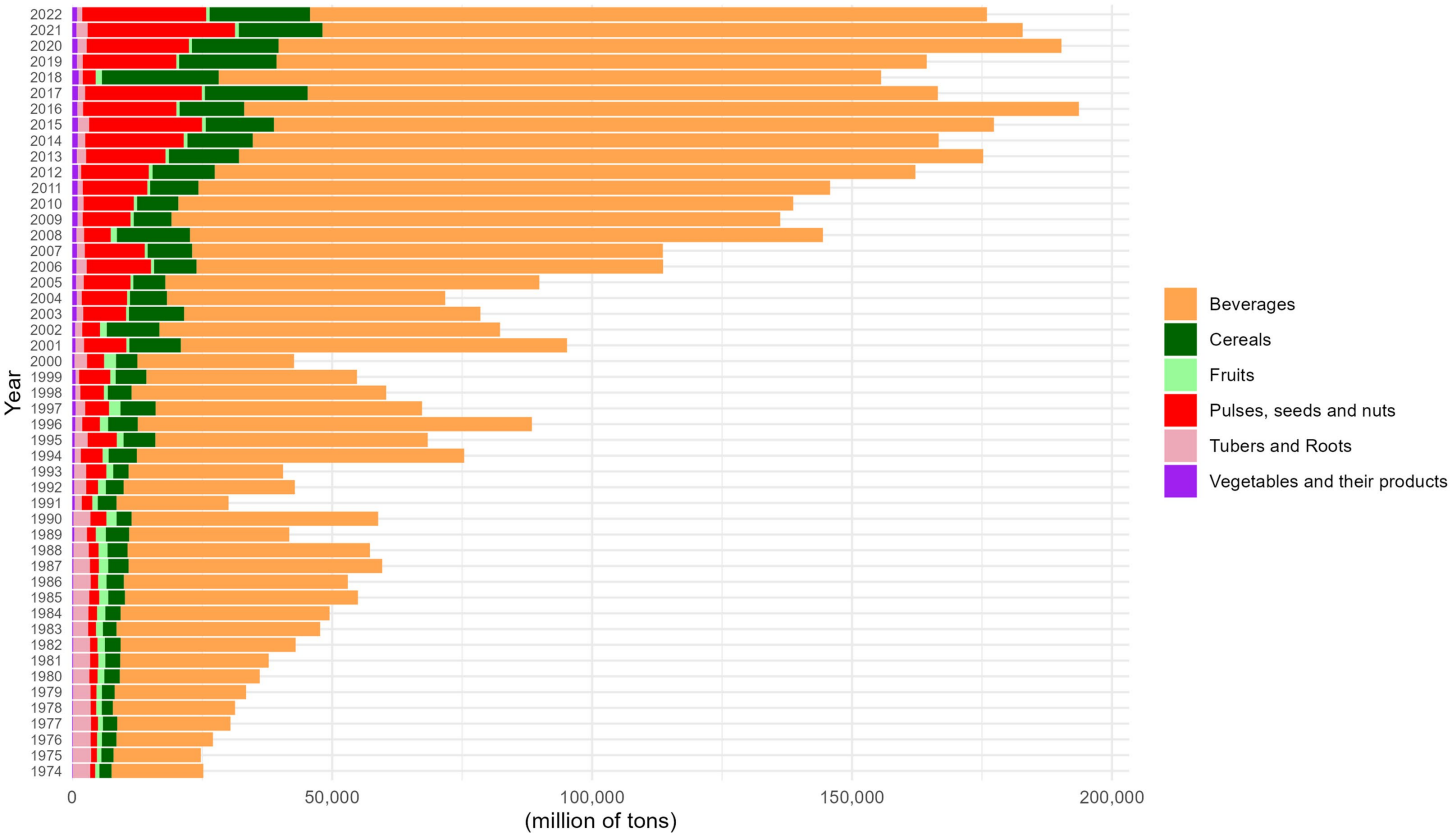
Trends in average food production by group in Brazil (1974–2022). This figure illustrates the average production of plant-based food groups, aggregated by year. The values are based on the mean levels of food production, expressed in tons. To enhance readability, the data have been scaled down by a factor of 10^2^, and the y-axis represents the total food production in millions of tons.

Among macronutrient-rich food sources, sugarcane (from the Beverages group) was the primary carbohydrate source throughout the entire analysis period (1974–2022), with peak production volumes recorded in the 1970s and 1980s. The state of São Paulo stood out as the leading producer, particularly in 2012, 2013, and 2016 (Figure 4).

Regarding protein and lipid sources, soybeans (from the Pulses, Seeds, and Nuts group) were the predominant crop. Whereas for micronutrients, the highest values across all years were observed for vitamin C, magnesium, and calcium, primarily derived from oranges, wheat, and soybeans, respectively.

### 3.4 Registered pesticides

**Figure 5:**
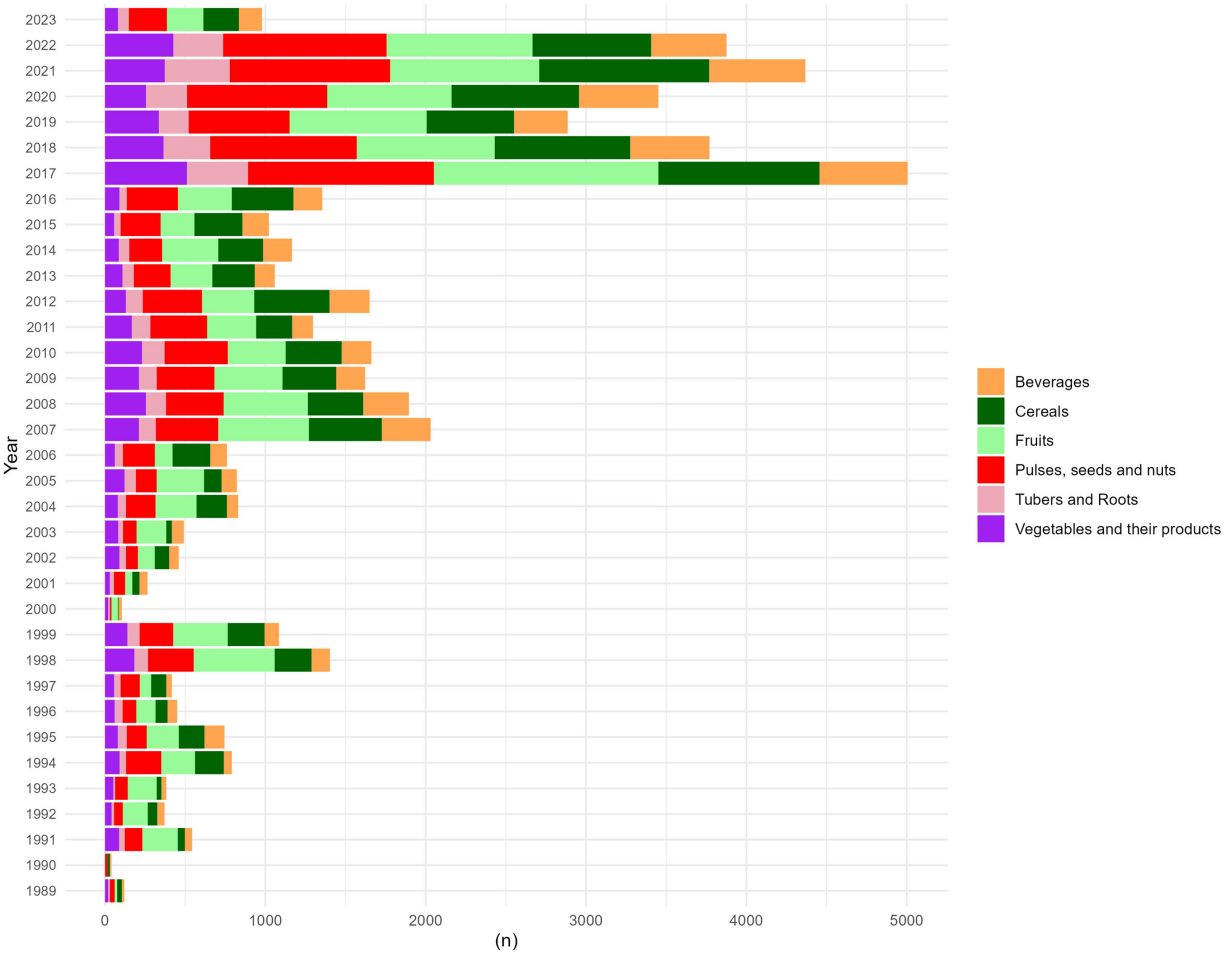
Number of pesticides registered across years. The color palette indicates the food groups associated with the number of pesticides registered across different years. Each color represents a specific food category: Fruits (Pale Green). Vegetables and Their Products (Purple). Tubers and Roots (Pink). Cereals (Green): Pulses, Seeds, and Nuts (Red). Beverages (Orange).

Between 2017 and 2022, there was a significant 338.90% increase in the total number of approved pesticides, rising from an annual average of 887 (±565) to 3,893 (±733) (Figure 5), covering all levels of environmental impact.

**Figure 6:**
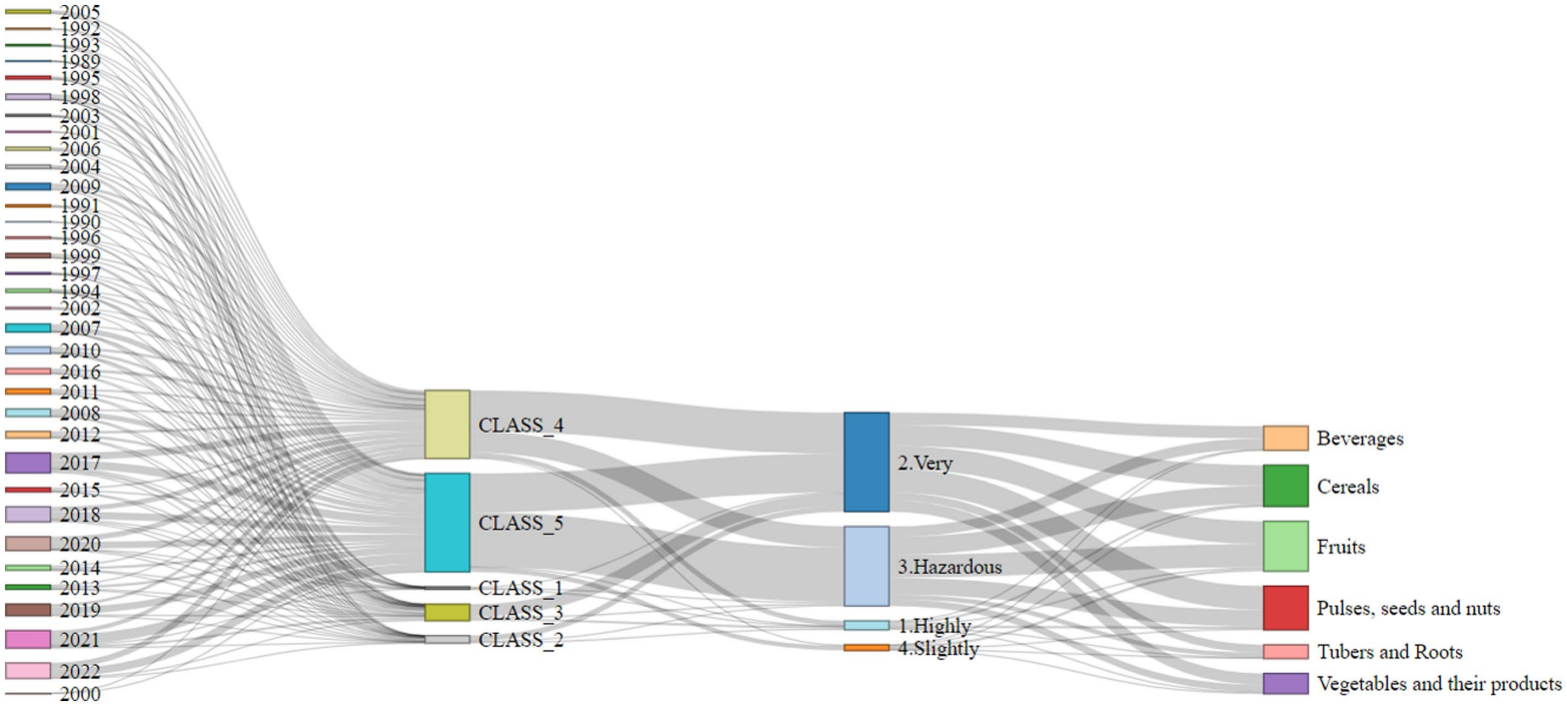
Diagram of pesticide registers across years, toxicological and environmental risk by food group. Toxicological Classification: Class 1 — Extremely Toxic; Class 2 — Highly Toxic; Class 3 — Moderately Toxic; Class 4 — Slightly Toxic; Class 5 — Product Unlikely to Cause Acute Harm. Environment risk level: (1) Highly Hazardous –significant risk to environmental health; (2) Very Hazardous — high risk to the environment; (3) Hazardous –moderate environmental impact; (4) Slightly Hazardous — low levels of environmental risk. The number of pesticides registered annually for different food items, grouped by their respective categories. Food groups are represented as follows (Table 1). An interactive version of this figure is available at: https://rpubs.com/MariaMiele/1255970

When analyzing the environmental impact classification, 2017 stood out as the year with the highest percentage of approvals for pesticides classified as “high” impact (13%) and “very high” environmental impact (17%), the highest figures recorded since 1989 (Figure 6).

### 3.5 Food and pattern consumption

**Figure 7:**
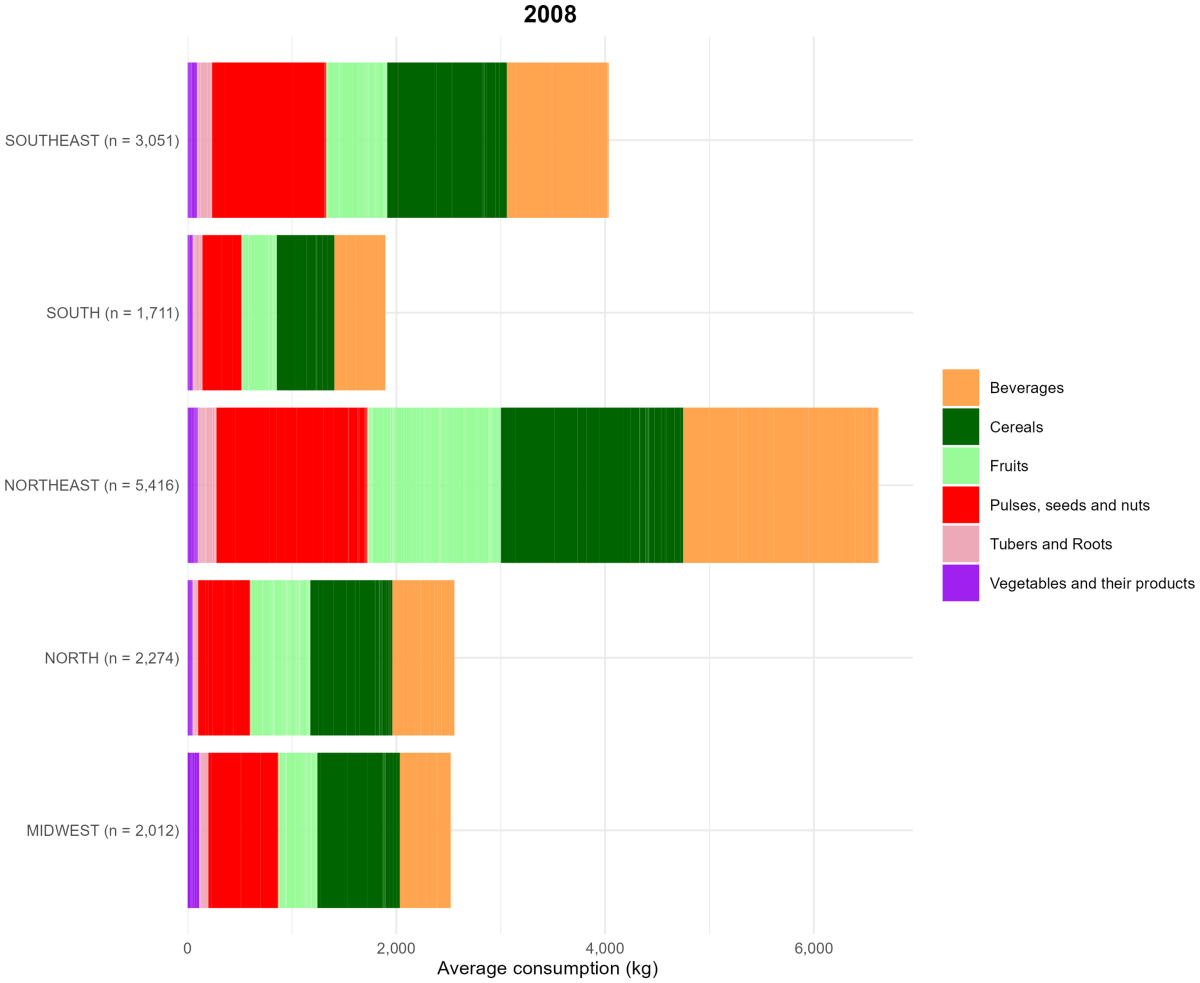
Food consumption (kg) by food groups, based on data from the Household Budget Survey (POF) 2008. Food groups are represented as follows (Table 1). The average food amount consumption (g) by region is described below: (1) Midwest = Beverages: 19.4%; Cereals: 31.3%; Fruits: 14.8%; Pulses, seeds and nuts: 26.6%; Tubers and Roots: 3.5%; Vegetables and their products: 4.4%. (2) North = Beverages: 23.3%; Cereals: 30.8%; Fruits: 22.5%; Pulses, seeds and nuts: 19.5%; Tubers and Roots: 2.1%; Vegetables and their products: 1.9%. (3) Northeast = Beverages: 28.3%; Cereals: 26.4%; Fruits: 19.3%; Pulses, seeds and nuts: 21.9%; Tubers and Roots: 2.7%; Vegetables and their products: 1.5%. (4) South = Beverages: 25.9%; Cereals: 29.1%; Fruits: 17.8%, Pulses, seeds and nuts: 19.8%, Tubers and Roots: 4.9%, Vegetables and their products: 2.5%. (5) Southeast = Beverages: 24.2%, Cereals: 28.4%, Fruits: 14.4%, Pulses, seeds and nuts: 27.1%, Tubers and Roots: 3.6%, Vegetables and their products: 2.2.

**Figure 8:**
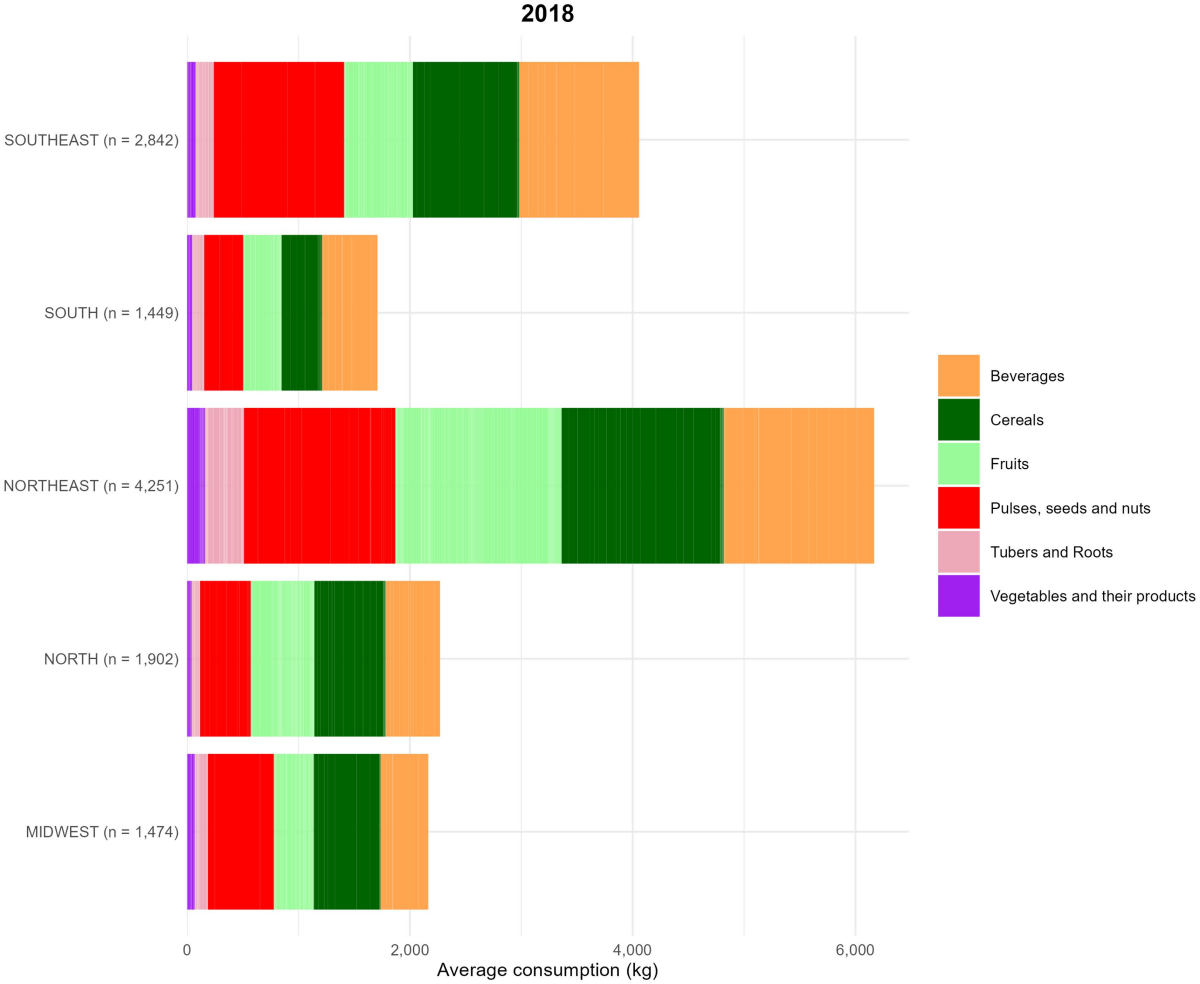
Food consumption (kg) by food groups, based on data from the Household Budget Survey (POF) 2018. Food groups are represented as follows (Table 1). The average food amount consumption (g) by region is described below: (1) Midwest = Beverages: 19.8%; Cereals: 27.7%; Fruits: 16.4%; Pulses, seeds and nuts: 27.4%; Tubers and Roots: 5.5%; Vegetables and their products: 3.1%. (2) North = Beverages: 21.5%; Cereals: 28.2%; Fruits: 25.1%; Pulses, seeds and nuts: 20.1%; Tubers and Roots: 3.3%; Vegetables and their products: 1.9%. (3) Northeast = Beverages: 21.9%; Cereals: 23.6%; Fruits: 24.2%; Pulses, seeds and nuts: 22.1%; Tubers and Roots: 5.6%; Vegetables and their products: 2.6%. (4) South = Beverages: 29.1%; Cereals: 21.3%; Fruits: 20.1%; Pulses, seeds and nuts: 20.6%; Tubers and Roots: 6.3%, Vegetables and their products: 2.6%. (5) Southeast = Beverages: 26.5%; Cereals: 23.6%; Fruits: 15.2%; Pulses, seeds and nuts: 28.9%; Tubers and Roots: 4%; Vegetables and their products: 1.9%.

The analysis of food consumption by group included a total of 26,382 women of reproductive age, with 11,918 participants in 2008 and 14,464 in 2018, from different regions of the country. In the assessment of the total quantity of food consumed by food groups across regions, in 2008, the highest percentage of consumption was attributed to the Beverages group in all regions, except in the Central-West, where cereals had the highest consumption. This pattern remained consistent in both evaluation periods (2008 and 2018) (Figures 7, 8).

However, when analyzing per capita food consumption among women, comparing patterns over a 10-year interval (2008–2018) (Tables 3, 4), significant changes were observed. Despite an increase in the number of participants, the average consumption per capita decreased between 2008 and 2018 for the Beverages, Cereals, and Fruits groups, whereas, in contrast, consumption increased for Pulses, Seeds and Nuts, Tubers and Roots, and Vegetables. Although the total per capita consumption remained unchanged, greater variability was observed, suggesting a possible substitution effect or diversification in dietary patterns over the decade.

**Table 3:** Distribution of food groups production and consumption (per capita) of women of reproductive age in 2008 by region.

POF 2008	Beverages (*N* = 1,088)	Cereals (*N* = 12,413)	Fruits (*N* = 796)	Pulse, seed, nut (*N* = 65)	Tuber, root (*N* = 91)	Vegetables (*N* = 11)	*p*-value
Food amount reported						< 0.001	
Mean (SD)	124.7 (82.2)	121.2 (63.7)	178.2 (122.9)	157.5 (97.9)	155.3 (119.1)	115.9 (120.0)	
Range	5.0–600.0	6.3–819.0	11.0–1290.0	17.0–420.0	16.0–600.0	30.0–400.0	
Region (%)						< 0.001	
Midwest	66 (6.1)	1885 (15.2)	54 (6.8)	2 (3.1)	4 (4.4)	1 (9.1)	
North	160 (14.7)	1820 (14.7)	283 (35.6)	4 (6.2)	7 (7.7)	0 (0.0)	
Northeast	540 (49.6)	4,539 (36.6)	259 (32.5)	40 (61.5)	34 (37.4)	4 (36.4)	
South	188 (17.3)	1,376 (11.1)	106 (13.3)	10 (15.4)	27 (29.7)	4 (36.4)	
Southeast	134 (12.3)	2,793 (22.5)	94 (11.8)	9 (13.8)	19 (20.9)	2 (18.2)	

Pesticides	Beverages (*N* = 1,685)	Cereals (*N* = 2,660)	Fruits (*N* = 4,140)	Pulse, seed, nut (*N* = 2,840)	Tuber, root (*N* = 920)	Vegetables (*N* = 1,745)	*p*-value
Toxicological classification (%)						< 0.001	
Class 1	50 (3.0)	160 (6.0)	0 (0.0)	115 (4.0)	0 (0.0)	10 (0.6)	
Class 2	35 (2.1)	60 (2.3)	95 (2.3)	70 (2.5)	30 (3.3)	40 (2.3)	
Class 3	75 (4.5)	215 (8.1)	135 (3.3)	180 (6.3)	60 (6.5)	95 (5.4)	
Class 4	535 (31.8)	725 (27.3)	1,625 (39.3)	990 (34.9)	325 (35.3)	660 (37.8)	
Class 5	990 (58.8)	1,500 (56.4)	2,285 (55.2)	1,485 (52.3)	505 (54.9)	940 (53.9)	
Environment Risk (%)						< 0.001	
Highly	55 (3.3)	135 (5.1)	115 (2.8)	135 (4.8)	40 (4.3)	90 (5.2)	
Very	810 (48.1)	1,260 (47.4)	2070 (50.0)	1,465 (51.6)	540 (58.7)	1,045 (59.9)	
Hazardous	770 (45.7)	1,225 (46.1)	1750 (42.3)	1,160 (40.8)	295 (32.1)	550 (31.5)	
Slightly	50 (3.0)	40 (1.5)	205 (5.0)	80 (2.8)	45 (4.9)	60 (3.4)	

Production	Beverages (*N* = 1,596)	Cereals (*N* = 6,567)	Fruits (*N* = 4,256)	Pulse, seed, nut (*N* = 5,882)	Tuber, root (*N* = 2,659)	Vegetable (*N* = 1,793)	*p*-value
Region (%)						< 0.001	
Midwest	228 (14.3%)	948 (14.4%)	628 (14.8%)	816 (13.9%)	427 (16.1%)	268 (14.9%)	
North	399 (25.0%)	1,638 (24.9%)	1,042 (24.5%)	1,450 (24.7%)	568 (21.4%)	448 (25.0%)	
Northeast	513 (32.1%)	2,133 (32.5%)	1,362 (32.0%)	1924 (32.7%)	775 (29.1%)	596 (33.2%)	
South	171 (10.7%)	690 (10.5%)	465 (10.9%)	656 (11.2%)	334 (12.6%)	190 (10.6%)	
Southeast	285 (17.9%)	1,158 (17.6%)	759 (17.8%)	1,036 (17.6%)	555 (20.9%)	291 (16.2%)	

Toxicological Classification: Class 1 — Extremely; Class 2 — Highly; Class 3 — Moderately; Class 4 — Slightly; Class 5 — Unlikely to Cause Acute Harm. Environment risk level: (1) Highly; (2) Very Hazardous; (3) Hazardous; (4) Slightly. The Kruskal-Walli’s test was used to assess variations in the per capita consumption of food groups among women across different regions. The Chi-square test was applied to examine the association between pesticide toxicological classifications, environmental risk levels, and regional distribution.

**Table 4:** Distribution of food groups production and consumption (per capita) of women of reproductive age in 2018 by region.

POF 2018	Beverages (*N* = 8,242)	Cereals (*N* = 2,302)	Fruits (*N* = 978)	Pulse (*N* = 104)	Tuber, root (*N* = 224)	Vegetables (*N* = 68)	*p*-value
Food amount reported (g)						< 0.001	
Mean (SD)	96.2 (100.1)	101.7 (64.7)	168.4 (94.7)	170.8 (94.4)	173.1 (142.4)	124.2 (132.6)	
Range	25.0–3000.0	12.5–1000.0	11.0–900.0	17.0–420.0	13.5–710.0	15.0–600.0	
Region (%)						< 0.001	
Midwest	939 (11.4)	371 (16.1)	127 (13.0)	14 (13.5)	11 (4.9)	12 (17.6)	
North	1,433 (17.4)	318 (13.8)	104 (10.6)	12 (11.5)	29 (12.9)	6 (8.8)	
Northeast	2,974 (36.1)	688 (29.9)	391 (40.0)	44 (42.3)	134 (59.8)	20 (29.4)	
South	872 (10.6)	364 (15.8)	159 (16.3)	20 (19.2)	23 (10.3)	11 (16.2)	
Southeast	2024 (24.6)	561 (24.4)	197 (20.1)	14 (13.5)	27 (12.1)	19 (27.9)	

Pesticide	Beverages (*N* = 4,125)	Cereals (*N* = 7,120)	Fruits (*N* = 8,970)	Pulse (*N* = 7,400)	Tuber, root (*N* = 2,280)	Vegetables (*N* = 3,685)	*p*-value
Toxicological classification (%)						< 0.001	
Class 1	75 (1.8)	230 (3.2)	10 (0.1)	165 (2.2)	5 (0.2)	30 (0.8)	
Class 2	150 (3.6)	355 (5.0)	215 (2.4)	330 (4.5)	135 (5.9)	135 (3.7)	
Class 3	285 (6.9)	640 (9.0)	565 (6.3)	785 (10.6)	285 (12.5)	335 (9.1)	
Class 4	1,475 (35.8)	2,435 (34.2)	3,375 (37.6)	2,720 (36.8)	830 (36.4)	1,500 (40.7)	
Class 5	2,140 (51.9)	3,460 (48.6)	4,805 (53.6)	3,400 (45.9)	1,025 (45.0)	1,685 (45.7)	
Environment risk (%)						< 0.001	
Highly	180 (4.4)	390 (5.5)	395 (4.4)	420 (5.7)	150 (6.6)	285 (7.7)	
Very	2015 (48.8)	3,610 (50.7)	4,200 (46.8)	4,185 (56.6)	1,400 (61.4)	2,150 (58.3)	
Hazardous	1850 (44.8)	3,050 (42.8)	3,950 (44.0)	2,655 (35.9)	660 (28.9)	1,120 (30.4)	
Slightly	80 (1.9)	70 (1.0)	425 (4.7)	140 (1.9)	70 (3.1)	130 (3.5)	

Production	Beverages (*N* = 1,596)	Cereals (*N* = 6,567)	Fruits (*N* = 4,256)	Pulse (*N* = 5,882)	Tuber, root (*N* = 2,659)	Vegetables (*N* = 1,793)	*p*-value
Region (%)						< 0.001	
Midwest	228 (14.3)	948 (14.4)	628 (14.8)	816 (13.9)	427 (16.1)	268 (14.9)	
North	399 (25.0)	1,638 (24.9)	1,042 (24.5)	1,450 (24.7)	568 (21.4)	448 (25.0)	
Northeast	513 (32.1)	2,133 (32.5)	1,362 (32.0)	1924 (32.7)	775 (29.1)	596 (33.2)	
South	171 (10.7)	690 (10.5)	465 (10.9)	656 (11.2)	334 (12.6)	190 (10.6)	
Southeast	285 (17.9)	1,158 (17.6)	759 (17.8)	1,036 (17.6)	555 (20.9)	291 (16.2)	

Toxicological Classification: Class 1 — Extremely; Class 2 — Highly; Class 3 — Moderately; Class 4 — Slightly; Class 5 — Unlikely to Cause Acute Harm. Environment risk level: (1) Highly; (2) Very Hazardous; (3) Hazardous; (4) Slightly. The Kruskal-Walli’s test was used to assess variations in the per capita consumption of food groups among women across different regions. The Chi-square test was applied to examine the association between pesticide toxicological classifications, environmental risk levels, and regional distribution.

In 2008, polished rice stood out as the primary source of plant-based macronutrients in women’s diets, contributing 81% of total protein intake, 82% of carbohydrates, and 74% of dietary fats. In 2018, white rice remained the most significant staple food, although its relative contribution decreased to 41% of proteins, 48% of carbohydrates, and 44% of fats. Beans, in 2008, ranked as the fifth most consumed food, providing 3% of proteins, 1% of carbohydrates, and 2% of fats. By 2018, its consumption increased, moving to the fourth position, with 7% of proteins, 2% of carbohydrates, and 4% of fats.

Soy milk made a minor contribution in 2008, accounting for 0.07% of proteins, 0.01% of carbohydrates, and 0.06% of fats. In 2018, protein (0.07%) and carbohydrate (0.01%) contributions remained unchanged, but fat content decreased to 0.02%. Similarly, sugarcane juice contributed with 0.04% of carbohydrate intake in 2008, decreasing to 0.02% in 2018.

To demonstrate the potential risk associated with food consumption (2008 and 2018) due to exposure to a variety of chemical agents, the number of registered pesticides was calculated for the period 1989–2008 (Table 3) and cumulatively from 1989 to 2018 (Table 4), based on toxicity and environmental risk classifications, distributed by food group. The number of chemical products classified as “Extremely Toxic” increased across all food groups. Regarding environmental risk, the “Very Hazardous” was predominant in the Pulses, Seeds, and Nuts group. Meanwhile, the “Hazardous” was the most representative across all food groups, increasing from 7,190 registered products in 2008 (51.4% of total registrations) to 17,560 in 2018 (52.3% of total registrations).

The assessment integrating production, consumption, and environmental impact revealed that, while the Central-West region was the largest producer of soybeans (Pulses, Seeds, and Nuts group), the diversity within this food group was more pronounced in the Northeast region. In 2008, the Northeast accounted for 32% of the total production of this group, a trend that persisted in 2018, with a 30% contribution.

## 4 Discussion

Our results link the historical geography of food production to the expansion of agricultural production and the growing number of registered pesticides, contrasting with dietary patterns and food security among women of reproductive age. This divergence raises potential risks that require further investigation.

This study revealed a disconnect between production and consumption. While staple crops such as rice and beans have historically shaped Brazilian diets, their cultivated areas declined after the 1990s. Meanwhile, from 2008 to 2018, per capita consumption of beans, tubers, roots, and vegetables increased, while beverages, cereals, and fruits declined—despite the prominence of these crops in agricultural production.

Brazil’s agricultural expansion has been driven by soybean and sugarcane cultivation, particularly in the Central-West and Southeast regions. Since 2001, this shift reflects changes in agricultural policies and frontier expansion, largely centered on soybean cultivation (21). São Paulo remains Brazil’s largest food producer, dominated by sugarcane - a crop whose colonial-era roots continue to shape its role in sugar-alcohol production, replacing coffee and other crops (22, 23).

Water use in soybean and sugarcane production highlights a major challenge: pressure on water resources. The water deficit is especially critical in dry years with low green water availability. From 2014 to 2017, Brazil faced severe droughts (24). While the Northeast suffered the greatest impact, its limited water infrastructure constrained large-scale agricultural water use. Conversely, the Southeast and Central-West maintained high consumption levels, supported by developed irrigation systems. The North follows a distinct pattern shaped by its climate, land use, and agricultural structure (Figure 1). These findings suggest that a few states account for the majority of agricultural water use, highlighting regional climate variability and infrastructure disparities.

Despite its high production volume, sugarcane contributes little to food security, as it is primarily destined for ethanol and sugar production (25–27). Similarly, soybean production reflects Brazil’s agricultural investment patterns, with most of it exported or used for animal feed, reinforcing its economic importance but limited direct contribution to human nutrition (28, 29).

The sharp increase in pesticide registrations signals a shift in regulatory patterns, allowing substances with high environmental and health risks. From 1991 to 2023, *glyphosate, 2,4-D dimethylamine, and trifluralin* - classified as “Highly Toxic” to human health and “Very Hazardous” to the environment - were authorized in Brazil, despite bans in other countries (30). *Glyphosate*, known as *Roundup*, is a widely used herbicide with documented airborne and dietary exposure risks. Studies suggest that glyphosate-based herbicides (GBHs) are even more harmful in combined formulations (31).

While pesticide authorizations continue to increase annually in Brazil, often including products with known toxicological risks, other regions have adopted different regulatory strategies. In Europe, regulatory frameworks such as the Farm to Fork Strategy aim to reduce pesticide use and promote agroecological transitions (32). Similarly, in North America, concerns about glyphosate exposure have led to litigation and regulatory reviews (33). While in 2023, tests on 5,068 food samples from different regions of Brazil found pesticide residues in one out of four foods, often exceeding the Maximum Residue Limit (MRL). Some foods contained multiple pesticide residues, raising concerns over chemical contamination in widely consumed products (34). While pesticides boost agricultural productivity, their application is largely self-regulated, with limited oversight on chemical usage and dosage (35).

Evidence of pesticide exposure in urine, blood, placenta, and breast milk suggests significant risks for female and reproductive health. The pharmacokinetics of pesticide exposure remain poorly understood, as do their transgenerational epigenetic effects, underscoring the urgent need for stricter exposure limits to protect human health and future generations (36).

Food is one of the major pathways of pesticide exposure, yet these exposures remain underreported (37, 38). A Brazilian study confirmed a link between pesticide use and adverse reproductive health outcomes, including fetal malformations, birth-related mortality, and small-for-gestational-age births (39). Research in rice and banana-growing regions detected pesticide metabolites in urine samples from pregnant women, demonstrating direct exposure to organophosphates and pyrethroids. This exposure correlated with lower birth weight and smaller head circumference in infants up to 1 year old (40). Reinforcing these warnings, further studies have demonstrated that exposure to certain pesticides during pregnancy can lead to adverse outcomes, such as reduced birth weight and smaller head circumference in infants (41).

Our study’s strength lies in its extensive timeframe and large-scale database integration, allowing us to examine agricultural shifts and their impact on dietary patterns among women of reproductive age. However, as a limitation, we did not include maternal and fetal health outcomes, as our primary focus was to analyze food production geography and historical trends. Future research should explore these health impacts in depth to assess potential long-term risks. Additionally, the results are subject to ecological inference and should be interpreted as population-level trends rather than causal relationships.

## 5 Conclusion

While rice and beans have long been staples of the Brazilian diet, providing an ideal combination of plant-based proteins for human consumption, soy has emerged as the dominant protein crop, occupying the largest planted area in the country. When evaluating the environmental and human health impacts of plant-based diets, our study highlights that this concept is far more complex than often assumed. The risk of contamination of soil, water, and especially food with toxic chemical residues calls into question the notion that plant-based diets are inherently healthier or more sustainable.

These findings underscore the need for a critical reassessment of plant-based food systems, considering the increasing number of authorized pesticides, which heighten the risk of contamination and pose threats to both environmental and human health. Future studies should explore health impacts on women of reproductive age and environmental effects such as biodiversity loss, land degradation, and greenhouse gas emissions.

In summary, beyond merely focusing on nutrient composition to shape public policies, food system management - from field to plate - must integrate food security and environmental sustainability as inseparable priorities. Ensuring a safe and healthy food supply requires a balanced approach that aligns agricultural practices with sustainable and responsible consumption.

## Data Availability

The dataset utilized in this study is available in an open-access repository at: https://doi.org/10.17632/mt4mj23j73.10. We adhere to the FAIR principles (Findable, Accessible, Interoperable, Reusable) to ensure that the data is well-organized, easily discoverable, and reusable.
