# Correction: From field to plate: 50 years of plant-based food production and emerging risks to planetary and women's health

**DOI:** 10.3389/fnut.2025.1666757

**Published:** 2025-08-04

**Authors:** Maria Julia Miele, Priscila Pereira Coltri, Camila Ferreira Soares, Renato Teixeira Souza, Rodolfo Carvalho Pacagnella, José Guilherme Cecatti, Barbara Teruel

**Affiliations:** ^1^School of Agricultural Engineering, University of Campinas (UNICAMP), Campinas, Brazil; ^2^Department of Obstetrics and Gynecology, School of Medical Sciences, University of Campinas (UNICAMP), Campinas, Brazil; ^3^Center for Meteorological and Climatic Research Applied to Agriculture (CEPAGRI), University of Campinas, Campinas, Brazil; ^4^Department of Demography, Institute of Philosophy and Human Sciences, University of Campinas (UNICAMP), Campinas, Brazil

**Keywords:** agri-food systems, dietary patterns, planetary health, pesticide residues, reproductive age

In the published article, there was an error in Figure 7 as published. Figure 7 (titled 2008) was inadvertently duplicated from Figure 8 (titled 2018). All numerical values, legends, and captions for Figures 7 and 8 are correct, only the image in Figure 7 should be replaced with the correct version corresponding to data from 2008.

The corrected Figure 7 image is provided below.

**Figure 7:**
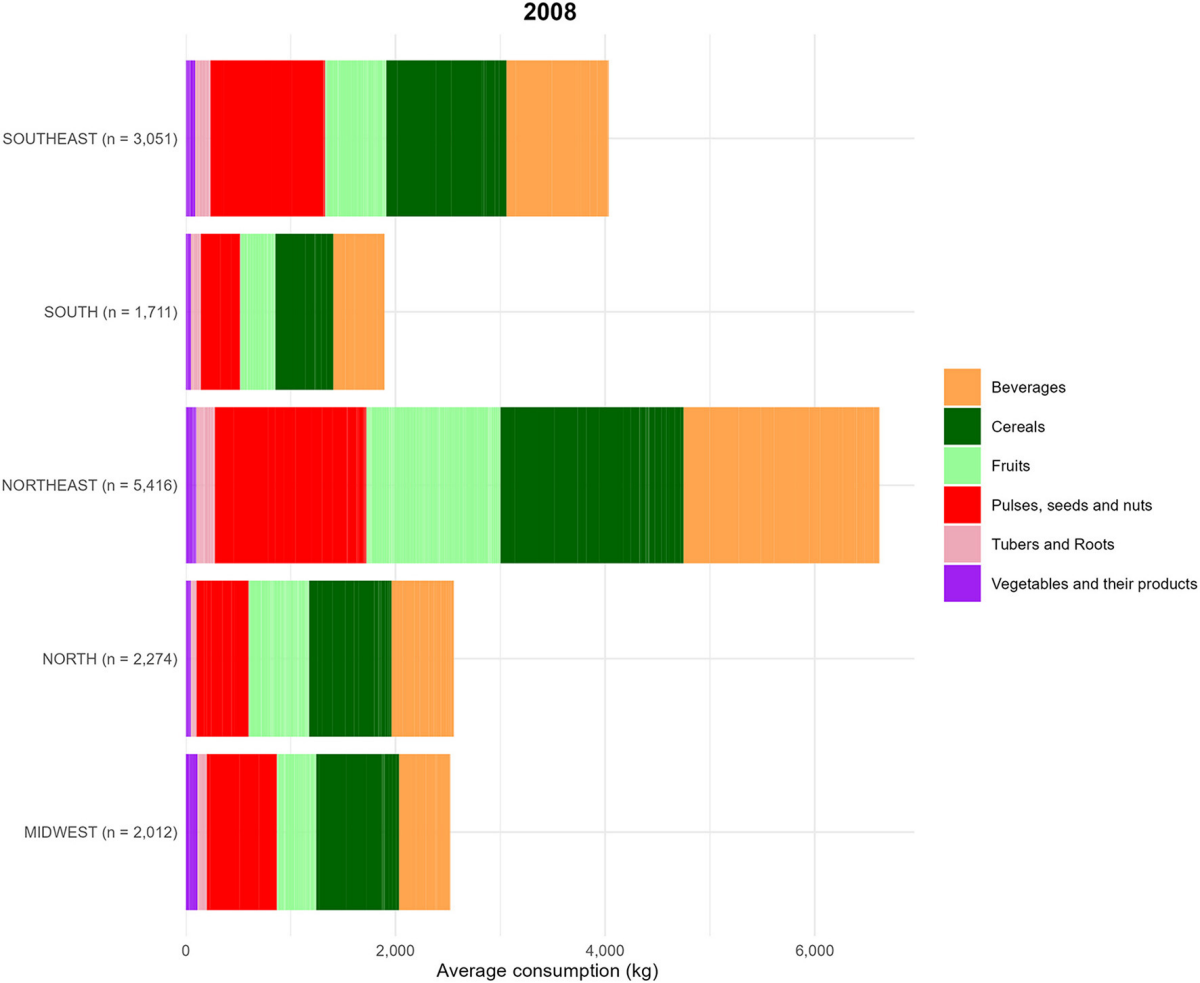
Food consumption (kg) by food groups, based on data from the Household Budget Survey (POF) 2008. Food groups are represented as follows (Table 1). The average food amount consumption (g) by region is described below: (1) Midwest = Beverages: 19.4%; Cereals: 31.3%; Fruits: 14.8%; Pulses, seeds and nuts: 26.6%; Tubers and Roots: 3.5%; Vegetables and their products: 4.4%. (2) North = Beverages: 23.3%; Cereals: 30.8%; Fruits: 22.5%; Pulses, seeds and nuts: 19.5%; Tubers and Roots: 2.1%; Vegetables and their products: 1.9%. (3) Northeast = Beverages: 28.3%; Cereals: 26.4%; Fruits: 19.3%; Pulses, seeds and nuts: 21.9%; Tubers and Roots: 2.7%; Vegetables and their products: 1.5%. (4) South = Beverages: 25.9%; Cereals: 29.1%; Fruits: 17.8%, Pulses, seeds and nuts: 19.8%, Tubers and Roots: 4.9%, Vegetables and their products: 2.5%. (5) Southeast = Beverages: 24.2%, Cereals: 28.4%, Fruits: 14.4%, Pulses, seeds and nuts: 27.1%, Tubers and Roots: 3.6%, Vegetables and their products: 2.2.

The original article has been updated.

